# Decreased Colonic Guanylin/Uroguanylin Expression and Dried Stool Property in Mice With Social Defeat Stress

**DOI:** 10.3389/fphys.2020.599582

**Published:** 2020-12-14

**Authors:** Nobuhiko Ebisutani, Hirokazu Fukui, Heihachiro Nishimura, Takashi Nakanishi, Kenki Morimoto, Shiho Itou, Ayumi Nakamura, Mizuki Masutani, Mika Hori, Toshihiko Tomita, Tadayuki Oshima, Emiko Kasahara, Atsuo Sekiyama, Hiroto Miwa

**Affiliations:** ^1^Division of Gastroenterology and Hapatology, Department of Internal Medicine, Hyogo College of Medicine, Nishinomiya, Japan; ^2^Department of Preemptive Medical Pharmacology for Mind and Body, Graduate School and School of Pharmaceutical Sciences, Osaka University, Suita, Japan

**Keywords:** social defeat stress, depression, constipation, guanylin, uroguanylin, guanylate cyclase 2C, glucocorticoid, stool

## Abstract

Psychological stress is deeply involved in the pathophysiology of not only mental illness but also functional gastrointestinal disorders. In the present study, we examined the relationship between psychological stress and abnormality of stool properties, focusing on the alteration of plasma glucocorticoid and guanylin (GN)/uroguanylin (UGN) expression in the colon. A murine model of chronic social defeat stress (CSDS) was established by exposing a C57BL/6N intruder mouse to a CD-1 aggressor mouse for 3–5 min. Thereafter the mice were kept in the same cage but separated by a divider for the remainder of the day. This procedure was repeated for 10 consecutive days, and then a social interaction test was performed to evaluate social avoidance. Fresh fecal and blood samples were collected for stool property analysis and measurement of the plasma glucocorticoid level by ELISA. The expression of GN, UGN, and guanylate cyclase 2C in the colonic tissues was examined by real-time RT-PCR and immunohistochemistry. Moreover, Lovo cells were stimulated with dexamethasone, and the expression of *GN*/*UGN* mRNA was examined. In the CSDS group, the time spent in the social interaction zone was significantly shorter when the CD-1 aggressor mouse was present than when it was absent. The social interaction ratio was also significantly lower in the CSDS group relative to the controls. The mean Bristol scale score was significantly lower in the CSDS group, but the fecal sodium concentration did not differ between CSDS mice and controls. The level of plasma corticosterone was significantly higher in the CSDS group than in the controls immediately after the 10th day of CSDS. The expression of both *GN* and *UGN* was significantly decreased in the CSDS mice. GN was expressed in all colonic epithelial cells, and UGN was expressed in ovoid or pyramidal epithelial cells in the colonic mucosa. The expression of both *GN* and *UGN* was significantly decreased in the CSDS mice relative to controls. The expression of both GN and UGN was significantly suppressed in Lovo cells upon stimulation with dexamethasone. Psychological stress-induced glucocorticoid may suppress colonic GN/UGN expression, resulting in a change in stool properties leading to constipation.

## Introduction

Psychological stress is deeply involved in the pathophysiology of not only mental illness but also systemic organ disease (Iwata et al., 2013). In the gastrointestinal tract, psychological stress is a key factor involved in the development of functional gastrointestinal disorders (FGIDs), which are characterized by symptoms such as abdominal pain, dysmotility or abnormality of stool properties in the absence of organic disease (Sibelli et al., 2016). Indeed, it is well known that patients with FGIDs show significantly higher anxiety scores (Fond et al., 2014; Enck et al., 2016). Moreover, it is interesting that patients with depression suffer from constipation very frequently (Garvey et al., 1990; Chattat et al., 1997; Cheng et al., 2003). However, the relationship between psychological stress and abnormality of stool properties remains unclear.

The functions of the gastrointestinal tract include not only the digestion/absorption of energy sources but also the secretion/re-absorption of ions and water (Cooke, 1989; Dubreuil, 2012; Laforenza, 2012). These functions are indispensable for systemic homeostasis and finely tuned by interaction between the brain and the gut (Cussotto et al., 2018). In this brain/gut axis, gut hormones are known to play a pivotal role as mediators (Cussotto et al., 2018). In this context, we have focused on the gut hormones guanylin (GN) and uroganylin (UGN), which promote fluid secretion into the intestinal lumen from intestinal epithelial cells by activating their receptor, guanylate cyclase 2C (GC-C; Nakazato, 2001). In the present study, to clarify the effect of psychological stress on stool properties, we investigated GN and UGN expression in mice subjected to chronic social defeat stress (CSDS), which is recognized to be a model of depression (Golden et al., 2011). Moreover, we examined the mechanism by which psychological stress inhibits GN/UGN expression and alters the properties of stools.

## Materials and Methods

### Murine Model of Chronic Social Defeat Stress

C57BL/6N and CD-1 mice were purchased from Japan SLC, Inc. (Hamamatsu, Japan). C57BL/6N mice were used at 6–7 weeks of age. The CD-1 mice, used as aggressors, were sexually experienced retired breeders at least 4 months of age. The mice were maintained under specific pathogen-free conditions in an environmentally controlled clean room with a 12 h light/12 h dark schedule, and allowed access to standard laboratory chow (CRF-1, Oriental Yeast Co., Ltd., Tokyo, Japan) and water *ad libitum*. All animal experiments were performed in accordance with the Osaka University guidelines for animal experiments. An experimental C57BL/6N intruder mouse was subjected to defeat stress by exposure to a CD-1 aggressor mouse as described previously (Golden et al., 2011) with minor modification. We modified the protocol to avoid fetal injury of an intruder mouse. Detail methods for the development of CSDS model was described in Supplementary Figure 1. Briefly, a C57BL/6N intruder mouse and a CD-1 aggressor mouse was kept in the same cage separated by a divider. Once a day, a C57BL/6N mouse was exposed to the defeat by a CD-1 mouse for 3–5 min, and thereafter, both mice were kept again in the same cage but separated by a divider for the remainder of the day. This procedure was repeated for 10 consecutive days using an aggressor CD-1 mouse every day. The body weight, food intake and water intake were recorded during the experiment.

### Social Interaction Test

The social interaction test was performed on the day after the completion of CSDS exposure as described previously (Golden et al., 2011) with minor modification. The behaviors of experimental mice were recorded using video camera throughout the test. Briefly, mice were placed in an open field box for two continuous 3-min sessions, the first one with the CD-1 mouse absent followed by a continuous second session with the CD-1 mouse present in a plexiglas container. All the mice were habituated in the absence of the CD-1 mouse for 5 min prior to the social interaction test. The social interaction ratio (SIR) was calculated by dividing the period of time attached the interaction zone with the CD-1 mouse present by the period of time attached the interaction zone with the CD-1 mouse absent. In the present study, we determined the mice as susceptible to CSDS when SIR is less than 40. All of the mice used in this study were susceptible to CSDS.

### Sampling of Stools, Blood and Colonic Tissues

On experimental day 11, fecal samples were collected from mice immediately after defecation and weighted. The property of the stools was scored according to the Bristol scale (Lewis and Heaton, 1997). The fecal samples were suspended at 0.03 mg/μl in deionized water. After centrifugation, the concentration of sodium ions in the supernatant was measured using a LAQUAtwin sodium ion meter (Horiba Scientific, Kyoto, Japan).

Mice were euthanized by cervical dislocation under sevoflurane anesthesia. Blood was obtained from the inferior vena cava into an EDTA-containing test-tube, and plasma was isolated by centrifugation (10000 × *g* for 10 min) and stored −80°C until ELISA assay. The colonic tissues were removed, cut open along the longitudinal axis, and stored in nitrogen liquid for RNA extraction or fixed in phosphate-buffered 10% formalin for immunohistochemistry.

### Cell Culture and Reagents

The human colon cancer cell line Lovo was cultured in RPMI 1640 medium (Invitrogen, Carlsbad, CA, United States) with 10% fetal bovine serum (Biowest, Nuaillé, France) in a humidified incubator at 37°C with an atmosphere of 5% CO2. As mentioned in Figure Legends, the cells were treated with recombinant human dexamethasone (Sigma-Aldrich, St Louis, MO, United States) at the indicated doses.

### Immunohistochemistry

Immunohistochemical staining for GN, UGN, and GC-C was performed with an Envision Kit (Dako, Kyoto, Japan) as described previously (Yang et al., 2017), using anti-GN antibody (dilution; 1:50; LSBio, Seattle, WA, United States), anti-UGN antibody (dilution; 1:500; Proteintech, Rosemont, IL, United States) and anti-GC-C antibody (dilution; 1:50; Biorbyt, St Louis, MO, United States). In brief, the rehydrated sections were treated by microwave heating for 20 min in Antigen Unmasking Solution (Vector Laboratories, Burlingame, CA, United States) for GN and GCC or in Dako Target Retrieval Solution pH 9 (Dako Denmark, Glostrup, Denmark) for UGN. The sections were then preincubated with 0.3% H2O2 in methanol for 20 min at room temperature to quench endogenous peroxidase activity. They were then incubated with primary antibodies for 60 min at room temperature, washed in PBS, and incubated with horseradish peroxidase-conjugated secondary antibody for 30 min. The slides were visualized using 3,3’-diaminobenzidine tetrahydrochloride with 0.05% H2O2 for 3 min and then counterstained with Mayer’s hematoxylin. The number of UGN-positive cells was counted in well-oriented perpendicular glands in at least seven different fields in each animal. The data were expressed as the number of positive cells per gland.

### Real-Time RT-PCR

Total RNA was isolated from GI tissues and Lovo cells with Trizol reagent (Invitrogen, Carlsbad, CA, United States). One microgram of total RNA was reverse-transcribed using an oligo-dT primer (Applied Biosystems, Branchburg, NJ, United States), and real-time RT-PCR was performed using a 7900H Fast Real-Time RT-PCR System (Applied Biosystems) as reported previously (Xu et al., 2019). The set of primers used is shown in Table 1. Real-time RT-PCR assays were carried out with 200 ng of RNA equivalent cDNA, SYBR Green Master Mix (Applied Biosystems), and 500 nmol/l gene-specific primers. The PCR cycling conditions were 50°C for 15 s and 60°C for 60 s. The intensity of the fluorescent dye was determined, and the expression levels of target gene mRNAs were normalized to those of *GAPDH* mRNA.

**TABLE 1:** Primers for real-time RT-PCR analysis.

Gene	Direction	Primer Sequence
*Mouse-guanylin*	Forward	5′-GATCCTGCAGAGGCTAGAGG-3′
	Reverse	5′-AAGGCAAGCGATGTCACTCT-3′
*Mouse-uroguanylin*	Forward	5′-AGGAGATGTCCAATCCCCAG-3′
	Reverse	5′-ACAGTTCACATTCGTCGGTGG-3′
*Mouse-guanylate cyclase 2C*	Forward	5′-TGTGAACGCGACTTTCATCTAC-3′
	Reverse	5′-GCAGCCCATCTTATGATCTCTTG-3′
*Mouse-GAPDH*	Forward	5′-GGAGAAACCTGCCAAGTATG-3′
	Reverse	5′-TGGGAGTTGCTGTTGAAGTC-3′

### Statistical Analysis

Statistical analyses were performed using StatView 5.0 software (SAS Institute, Inc., Buckinghamshire, United Kingdom). All values were expressed as the mean ± SE. Significance of differences between two animal groups was analyzed by Mann–Whitney *U*-test. Differences were considered to be significant at *p* < 0.05.

## Results

### Social Interaction Test

**FIGURE 1:**
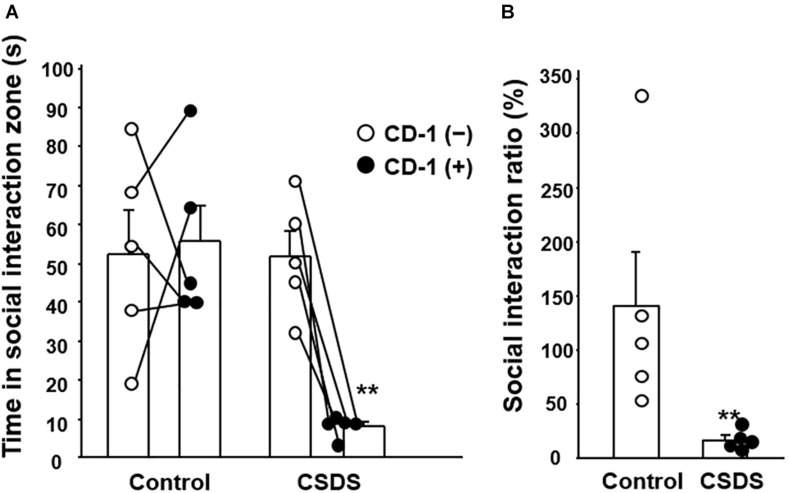
Effect of chronic social defeat stress (CSDS) on social interaction. **(A)** Time spent in the social interaction zone in the absence and presence of the CD-1 aggressor mouse. **(B)** Social interaction ratio calculated as the time spent in the social interaction zone in the presence of the CD-1 aggressor mouse divided by that in its absence. The social interaction rate in CSDS mice examined is less than 40. Results are expressed mean ± SE. Control mice (*n* = 5). CSDS mice (*n* = 5). Significantly smaller than in the controls: **P < 0.01.

To evaluate the effect of CSDS on the development of social avoidance, the social interaction test was employed. In the control group, the time spent in the social interaction zone did not differ between the situations with and without the CD-1 aggressor mouse. However, in the CSDS group, the time spent in the social interaction zone was significantly shorter when the CD-1 aggressor mouse was present (8.0 ± 1.3 s) than when it was absent (51.6 ± 6.6 s) (Figure 1A). Subsequently, the SIR was significantly lower in the CSDS group (16.7 ± 4.1%) relative to the controls (140.1 ± 50.9%) (Figure 1B).

### General Condition and Stool Properties in Mice With CSDS

**FIGURE 2:**
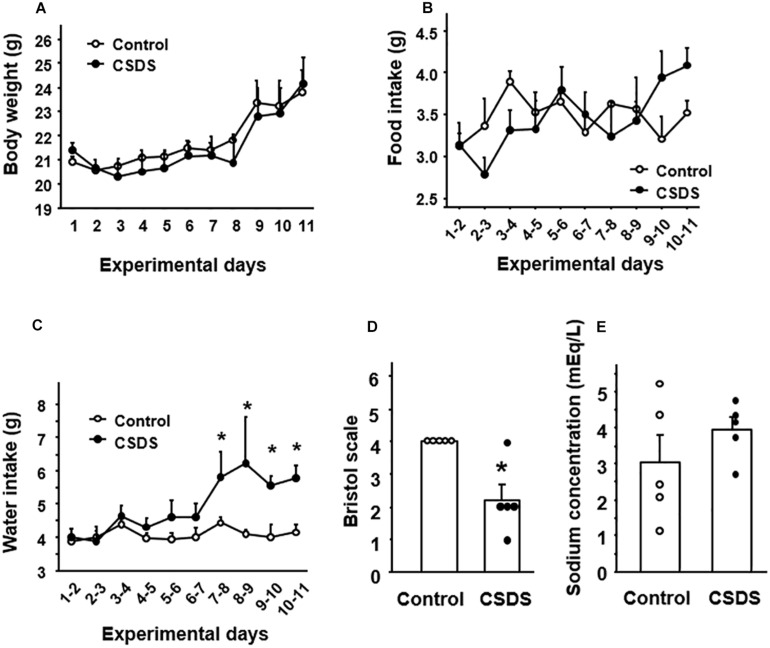
General condition and stool properties in mice with chronic social defeat stress (CSDS). Change of **(A)** body weight, **(B)** food intake, and **(C)** water intake. **(D)** Stool form according to the Bristol scale. **(E)** Stool sodium concentration. The social interaction rate in CSDS mice examined is less than 40. Results are expressed as the mean ± SE. Control mice (*n* = 5). CSDS mice (*n* = 5). Significantly different from the controls: *P < 0.05.

Body weight and food intake did not differ between controls and CSDS mice statistically (Figures 2A,B). Water intake was significantly greater in CSDS mice after experimental day 7 (Figure 2C). All of the control mice defecated stools with an apparently normal shape and moistness appear (Bristol scale score 4). On the other hand, the mean Bristol scale score was significantly lower in the CSDS group (2.2 ± 0.5), suggesting a loss of stool moisture (Figure 2D). Preliminary data of fecal water content was showed in Supplementary Figure 3.

Evaluation of the sodium ion content of the stools revealed no significant difference in fecal sodium concentration between CSDS mice and controls (Figure 2E).

### Plasma Corticosterone

**FIGURE 3:**
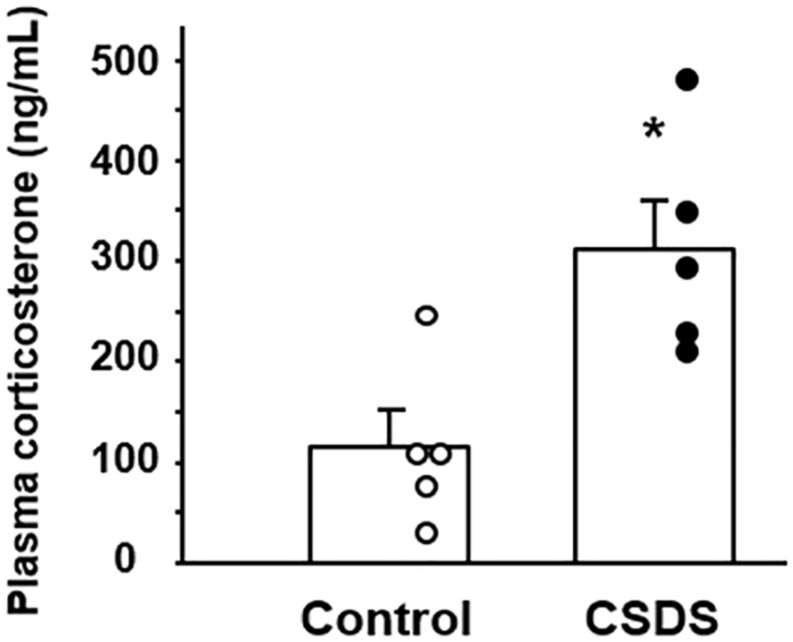
Plasma corticosterone concentrations in mice with chronic social defeat stress (CSDS). Control mice (*n* = 5). CSDS mice (*n* = 5). The social interaction rate in CSDS mice examined is less than 40. Results are expressed as the mean ± SE. Significantly higher than in the controls: *P < 0.05.

The plasma corticosterone level was analyzed to assess the activity of the hypothalamus-pituitary–adrenal axis. The level of plasma corticosterone was significantly higher in the CSDS group (312.6 ± 48.8 ng/ml) than in the controls (115.0 ± 35.9 ng/ml) (Figure 3).

### Expression of Guanylin, Uroguanylin and GC-C in the Colonic Tissues of Mice With CSDS

**FIGURE 4:**
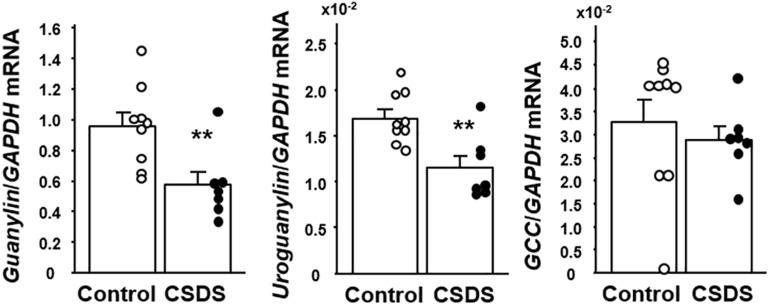
Colonic expression of guanylin, uroguanylin and guanylate cyclase 2C (GC-C) mRNA in mice with chronic social defeat stress (CSDS). Control mice (*n* = 9). CSDS mice (*n* = 7). The social interaction rate in CSDS mice examined is less than 40. Results are expressed as the mean ± SE. Significantly lower than in the controls: **P < 0.01.

We first examined the expression of GN, UGN and GC-C mRNAs in the colonic tissues of the experimental mice (Figure 4). The expression of both *GN* and *UGN* was significantly decreased in the CSDS mice relative to the controls. On the other hand, the expression of GC-C did not differ between the controls and the CSDS mice.

**FIGURE 5:**
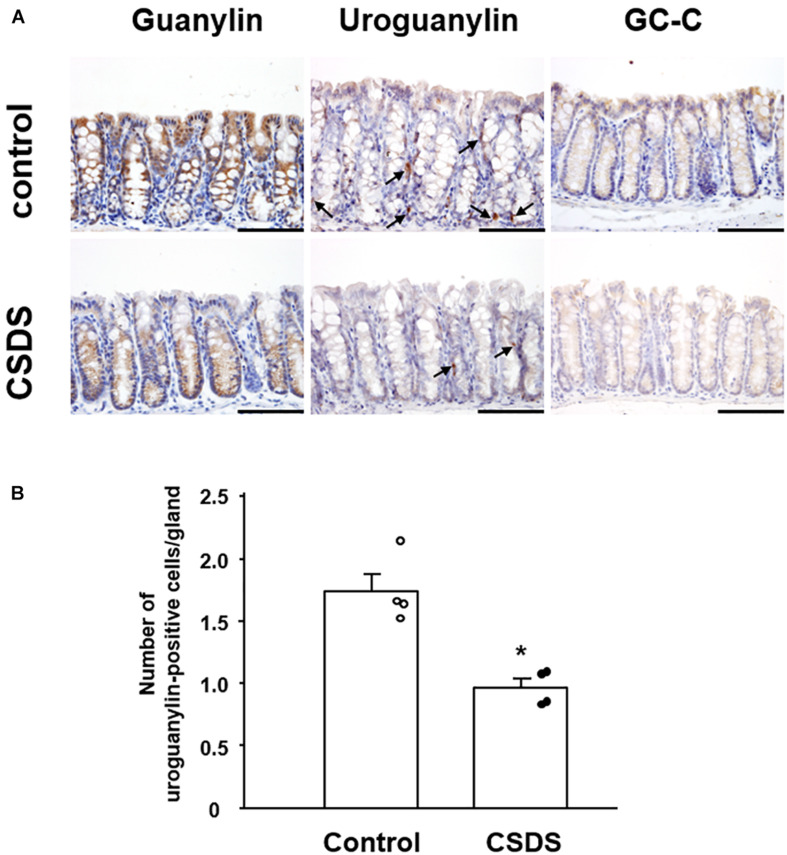
Effect of chronic social defeat stress (CSDS) on guanylin, uroguanylin and guanylate cyclase 2C (GC-C) in the colonic mucosa. **(A)** Immunostaining of uroguanylin and guanylate cyclase 2C (GC-C) in the colonic mucosa. Arrows indicate uroguanylin-positive cells. **(B)** The number of uroguanylin-positive cells in the colonic epithelium. Bar = 100 μm. Control mice (*n* = 4). CSDS mice (*n* = 4). The social interaction rate in CSDS mice examined is less than 40. Results are expressed as the mean ± SE. Significantly smaller than in the controls: *P < 0.05.

We next examined the localization of immunoreactivity for GN, UGN, and GC-C in the colonic tissues of the experimental mice (Figure 5A). GN was expressed in the cytoplasm of epithelial cells in the entire colonic mucosal layer, and its immunoreactivity appeared to be weaker in CSDS mice than in controls. UGN immunoreactivity was detected in the cytoplasm of ovoid or pyramidal epithelial cells in the colonic mucosa, a staining pattern that was morphologically compatible with endocrine cells (Figure 5A). The number of colonic UGN-positive cells was significantly decreased in CSDS mice relative to the controls (Figure 5B). GC-C immunoreactivity was detected in epithelial cells in the entire colonic mucosal layer, and appeared to be expressed similarly in both controls and CSDS mice (Figure 5A).

### Effect of Dexamethasone on Guanylin/Uroguanylin Expression in Lovo Cells

**FIGURE 6:**
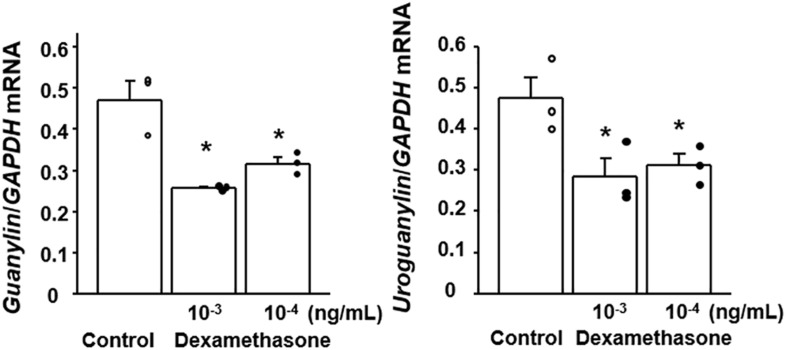
Effect of dexamethasone on the expression of guanylin and uroguanylin mRNAs in Lovo cells. Lovo cells (2 × 10^5^) were cultured in six-well plates and then treated with dexamethasone at the indicated concentrations for 72 h. Results are expressed as the mean ± SE (*n* = 3 in each group). Significantly smaller than in the controls: *P < 0.05.

Since plasma corticosterone was found to be increased in the CSDS mice, we examined the effect of dexamethasone on the expression of GN and UGN in colonic epithelial cells *in vitro*. We screened the expression of GN and UGN in several colon cancer cell lines and found that Lovo cells expressed both GN and UGN (data not shown). When the Lovo cells were stimulated with dexamethasone, the expressions of *GN* and *UGN* was significantly suppressed (Figure 6).

## Discussion

To validate whether the CSDS model had been adequately established, we first evaluated social avoidance in the CSDS mice using the social interaction test. It has been known that most of mice are susceptible to CSDS but 10–30% of mice are resilient (Golden et al., 2011). In this regard, we used CSDS susceptible mice in the present study. In line with previous reports (Huang et al., 2016), the CSDS mice examined were confirmed to show significant anxiety behavior, indicating that they were suitable as a depression model. It has been widely accepted that psychological stress is deeply involved in the pathophysiology of gastrointestinal functional disorders (Iwata et al., 2013). For instance, maternal separation stress cause diarrhea and acceleration of mucosal permeability (Fukui et al., 2018; Wong et al., 2019), which are observed in patients with irritable bowel syndrome. Interestingly, we found that Bristol scale for fecal humidity was significantly decreased in CSDS mice relative to controls. It has been reported that depressed patients often suffer constipation and fecal dryness. These findings suggest that the present murine CSDS model is useful for clarifying the pathophysiology of gastrointestinal functional disorders in depressed patients. On the other hand, accumulating evidences have suggested that not only psychological stress but also gut flora alteration has been closely involved in constipation (Ge et al., 2017). In this context, we preliminarily investigated the gut microbiota profile in CSDS susceptible mice and found that its profile is indeed different from that in controls (Supplementary Figure 2). However, we cannot exclude the contamination of bacterial strains from CD-1 aggressor mice to C57BL/6N intrude mice in this experiment although interesting reports have shown abnormal composition of gut microbiota in CSDS resilient mice and CSDS susceptible mice (Yang et al., 2017a,b).

The luminal contents of the small intestine are still fluid as they move to the colon. During formation of stools in the colon, their water content is determined by a balance of absorption and secretion during transport in the lumen. Here, we focused on GN and UGN in CSDS model mice because these gut hormones play a pivotal role in the regulation of water homeostasis in the colon (Nakazato, 2001). As shown in Figure 5, UGN was expressed in endocrine cells in the colonic mucosa, and GN was expressed in the entire colonic epithelium. One noteworthy finding was that GN and UGN expression was significantly decreased in the colonic tissues of CSDS mice. Since GN and UGN play help mediate the secretion of water into the intestinal lumen (Nakazato, 2001), this reduction of GN/UGN expression would appear to be well consistent with the reduced Bristol scale for fecal humidity in CSDS mice. Another noteworthy finding was that water intake was significantly increased in CSDS mice compared with controls. This may suggest that CSDS mice need hydration into body. In this context, the suppression of GN/UGN expression appears to be very reasonable. Thus, the suppression of GN/UGN leads to the inhibition of water secretion into the gastrointestinal lumen. Although we could not clarify whether CSDS mice showed rehydration, water balance may be the key to understand the alteration of stool property in CSDS mice.

However, the mechanism responsible for the reduction of colonic GN/UGN expression in CSDS mice has remained unclear. Previous studies have reported that dietary salt intake up-regulates the expression of GN/UGN (Carrithers et al., 2002), whereas low salt intake down-regulates this expression in the colon *in vivo* (Li et al., 1996). In addition, imposition of hypertonic stress using sodium chloride is able to enhance the expression of GN/UGN in colonic epithelial cells *in vitro* (Steinbrecher et al., 2002). These finding suggest that the luminal concentration of sodium and/or osmotic pressure may affect the degree of GN/UGN expression in the colon. In this context, we evaluated the sodium concentration of stools from experimental mice and observed no significant difference between controls and CSDS mice, suggesting that sodium concentration may not be involved in the reduced expression of colonic GN/UGN in this animal model. On the other hand, since the psychological stress in this model is certainly a key factor in the development of stool abnormality, we focused on corticosterone which is commonly upregulated in various stress models (Sudo et al., 2004; Ait-Belgnaoui et al., 2014; Jafari et al., 2017) and plays a pivotal role in the brain-gut axis (Shah et al., 2019). This revealed that the serum corticosterone level was indeed significantly increased in CSDS mice. We then examined the effect of dexamethasone stimulation on colonic epithelial cells *in vitro* and found that it significantly suppressed the expression of *GN*/*UGN*. These may suggest that corticosterone is able to suppress the transcription of *GN*/*UGN* genes. Interestingly, it is known that glucocorticoid represses AP-1 transcriptional activity via the glucocorticoid receptor, resulting in the suppression of target genes expression (Herrlich, 2001; Greenstein et al., 2002). In this regard, it has been clarified that the AP1 site is present in the promoter region of the *GN*/*UGN* genes and actually functions in their transcriptions (Hill et al., 1995; Miyazato et al., 1997). Accordingly, it is possible that dexamethasone may suppress GN/UGN expression in a similar manner in *in vitro*. Of course, we are unable to exclude the possibility that not only corticosterone but also other molecules might be involved in the suppression of GN/UGN expression, and that other mechanisms may play a role in reducing the water content of stools in CSDS mice. However, it may be interesting to speculate that accelerated corticosterone secretion is linked to the suppression of GN/UGN expression and a resulting reduction of water secretion into the colonic lumen, thus accounting for the lower fecal water content in CSDS mice.

In summary, we have shown that CSDS mice show disorders of not only behavior but also stool properties. This is consistent with the fact that patients with depression frequently suffer from constipation (Garvey et al., 1990; Chattat et al., 1997; Cheng et al., 2003). Although we are still unable to confirm the mechanism by which psychological stress affects the properties of stools and leads to constipation, we have shown here that CSDS mice have an increased plasma glucocorticoid level, and that their colonic GN/UGN expression is decreased. Moreover, we clarified that glucocorticoid stimulation suppresses the expression of *GN*/*UGN in vitro*. Together, it is tempting to speculate that psychological stress-induced glucocorticoid may affect water homeostasis-associated gut hormones, resulting in a change in fecal properties leading to constipation. On the other hand, we know that this study would need other *in vivo* studies using corticosterone administration and/or more precise *in vitro* studies to clarify the regulatory mechanism of corticosterone-associated suppression of GN/UGN expression. Thus, other possible mechanism cannot be excluded, this hypothesis warrants further investigation.

## Data Availability Statement

The original contributions presented in the study are included in the article/Supplementary Materials, further inquiries can be directed to the corresponding author/s.

## Ethics Statement

The animal study was reviewed and approved by Osaka University guidelines for animal experiments.

## Author Contributions

NE, HF, EK, and AS contributed to conceptualization. NE, HF, HN, TN, KM, SI, AN, MM, MH, and EK contributed to laboratory investigation, data acquisition and analysis. NE, HF, EK, and AS wrote the original draft of the manuscript. NE, HF, TT, TO, EK, AS, and HM contributed to review and editing of the manuscript. TT, TO, EK, AS, and HM contributed to supervision. All authors reviewed and approved the final version of the manuscript.

## Conflict of Interest

The authors declare that the research was conducted in the absence of any commercial or financial relationships that could be construed as a potential conflict of interest.
