# Supplementary figures and images for: Decreased Colonic Guanylin/Uroguanylin Expression and Dried Stool Property in Mice With Social Defeat Stress
Source: Front Physiol. 2020 Dec 14;11:599582. doi: 10.3389/fphys.2020.599582 (PMC7767843; doi:10.3389/fphys.2020.599582)

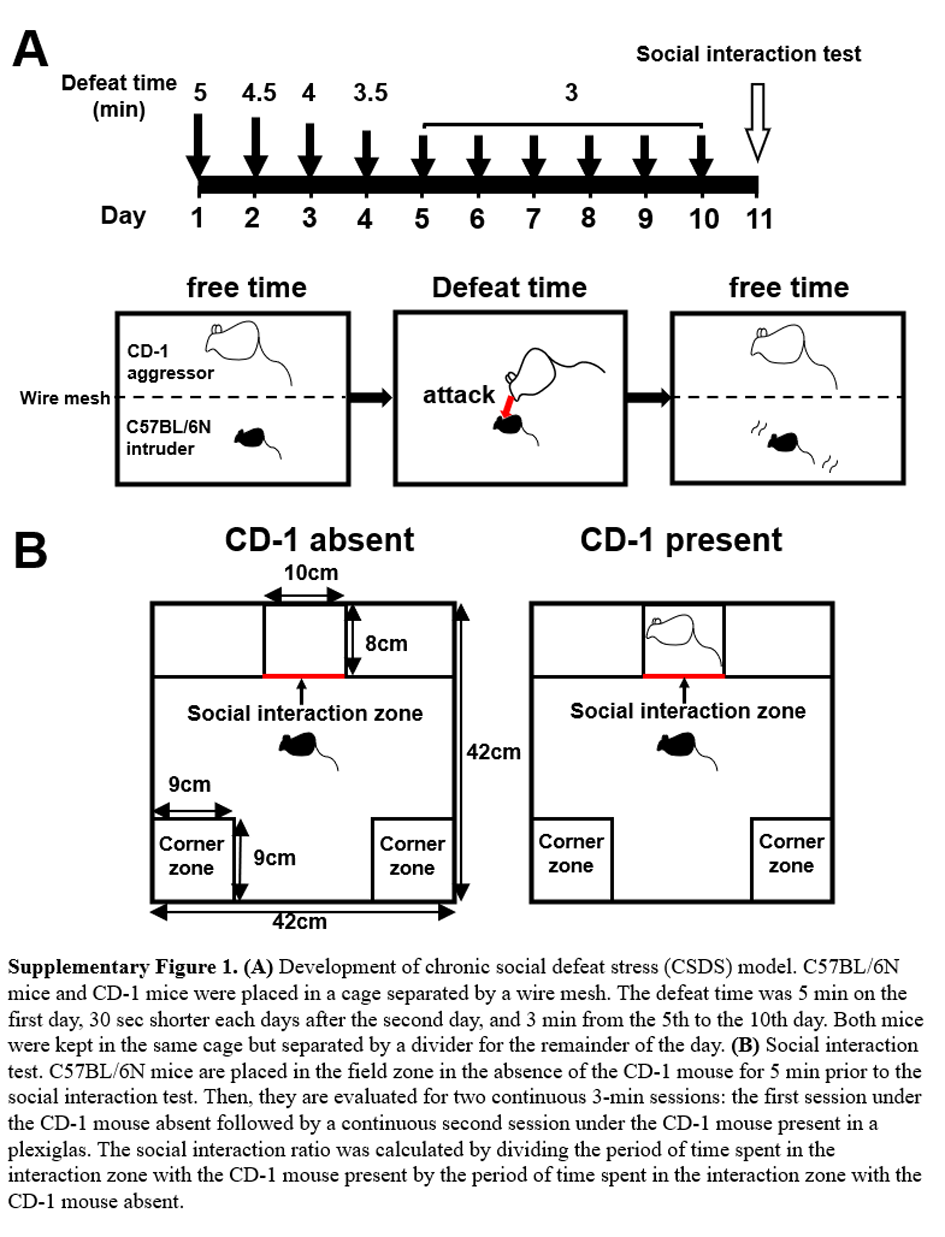

Supplement: Supplementary file 1 [file Image_1.TIF]

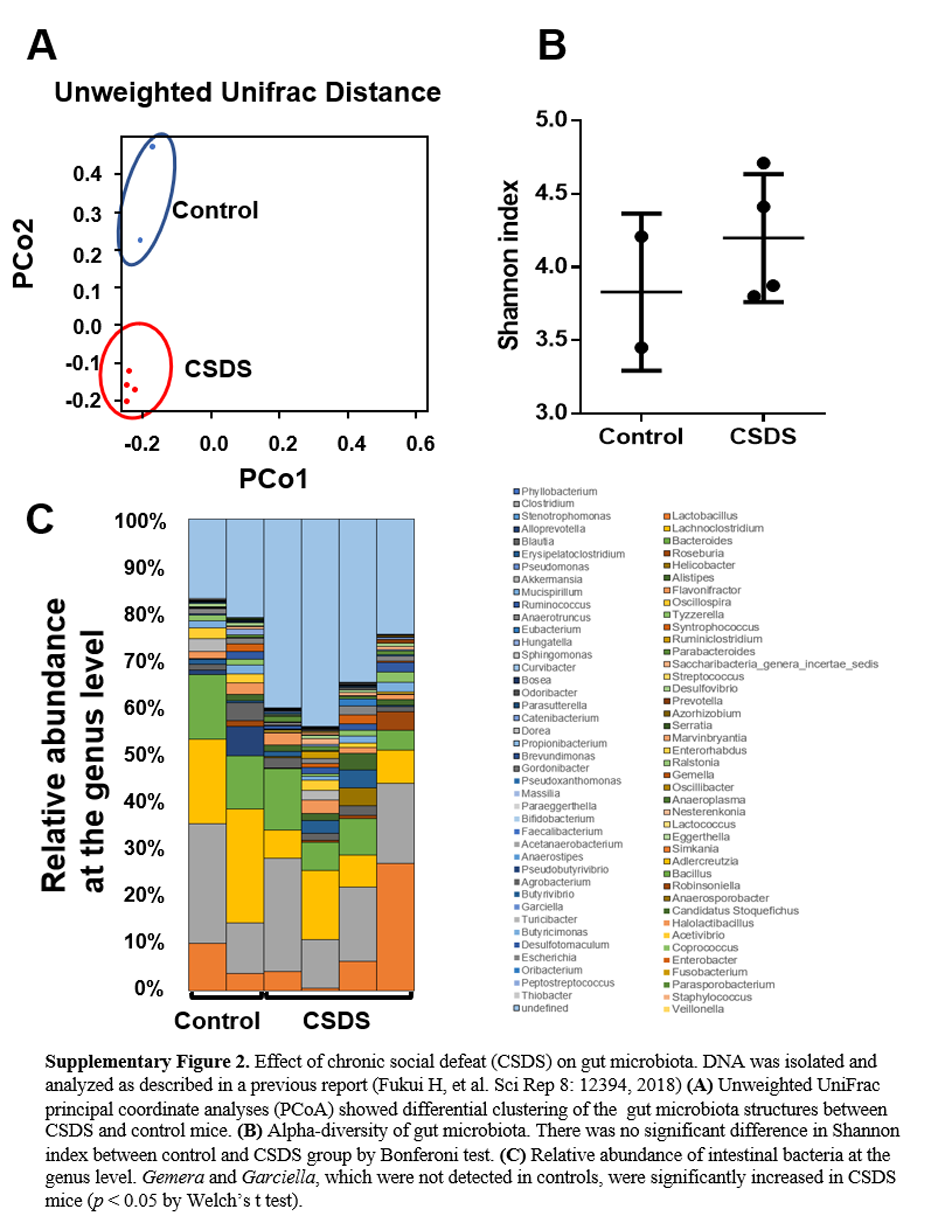

Supplement: Supplementary file 2 [file Image_2.tif]

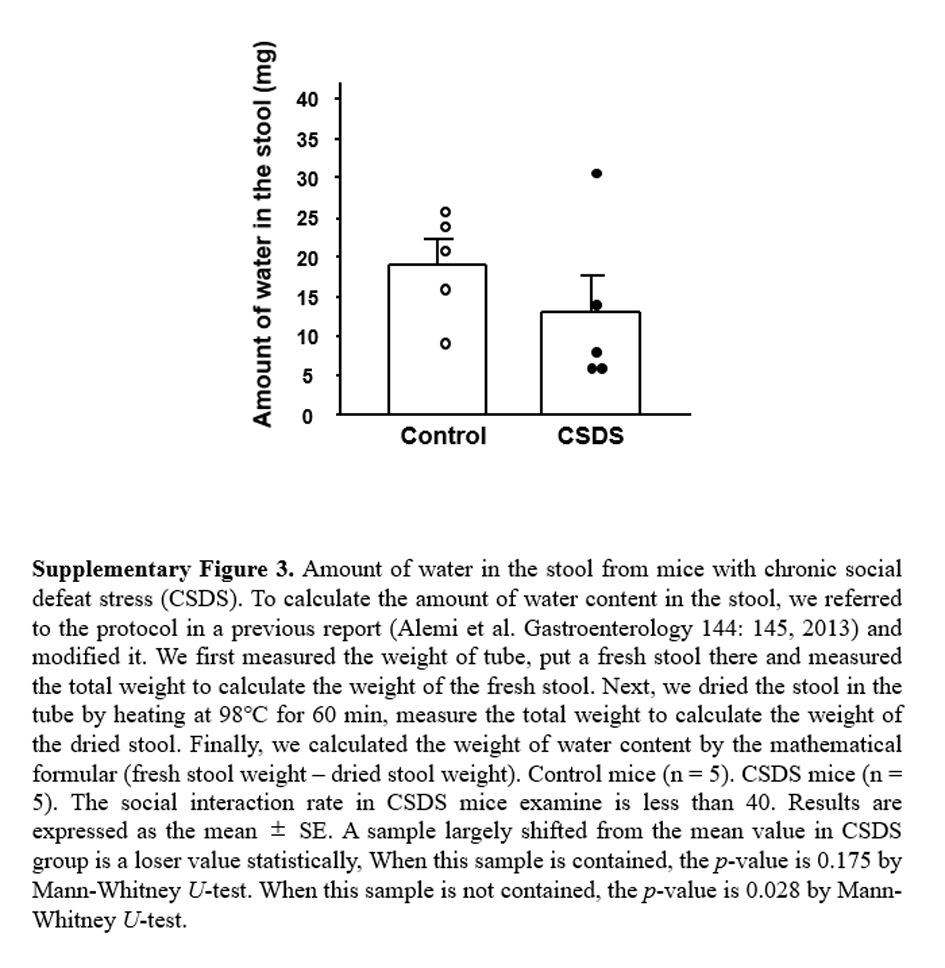

Supplement: Supplementary file 3 [file Image_3.tif]
